# Diagnosing bipolar disorders in DSM-5

**DOI:** 10.1186/2194-7511-1-14

**Published:** 2013-08-23

**Authors:** Emanuel Severus, Michael Bauer

**Affiliations:** Department of Psychiatry and Psychotherapy, University Hospital Carl Gustav Carus, Technische Universität Dresden, Fetscherstraße 74, Dresden, 01307 Germany

**Keywords:** Diagnosis, Bipolar disorders, DSM-5

## Editorial

A few weeks ago, after many years of intensive work, the much-awaited fifth edition of the Diagnostic and Statistical Manual of Mental Disorders (DSM-5) was published. It is still the case today that psychiatric diagnoses seem to be more consensus-based than validity-based (Cuthbert and Insel 2013; Bschor et al. 2012; Berk 2013) - something that DSM-5 will also be unable to change. In spite of this, DSM-5 introduces several important changes with regard to diagnostic criteria for bipolar disorders. The International Journal of Bipolar Disorders is honored that Jules Angst, whose work has made an outstanding contribution to the modifications regarding bipolar disorders in DSM-5 (Angst et al. 2011,2012), has agreed to comment on the strengths, problems and perspectives relating to these changes in the paper that accompanies this editorial (Angst 2013).

An essential topic thankfully addressed by Jules Angst in the accompanying paper (Angst 2013) has been hotly debated within the psychiatric scientific community throughout the last few years - namely whether bipolar disorders are much more frequent than previously assumed. If this is the case, one may conclude that the hitherto existing diagnostic criteria have falsely prevented the proper diagnosis of all cases of bipolar disorders on account of their being overly restrictive.

In DSM-5, bipolar and related disorders, as they are now called, are given a chapter on their own, between depressive disorders and schizophrenia spectrum disorders, that includes bipolar I disorder (which represents, according to DSM-5, classic manic depressive disorder, with the exception that neither a depressive episode nor psychosis has to be present for diagnosis), bipolar II disorder and cyclothymic disorder. Furthermore, in this chapter, there are now separate diagnostic criteria for “manic-like phenomena” associated with the use of substances (either substances of abuse or prescribed medications) or with medical conditions. Finally, to encourage further study, as the DSM-5 explicitly states, bipolar-like phenomena that do not fulfill the diagnostic criteria for bipolar I disorder, bipolar II disorder or cyclothymic disorder (i.e. short-duration hypomanic episodes and major depressive episodes, hypomanic episodes with insufficient symptoms and major depressive episodes, hypomanic episode without prior major depressive episode, and short-duration cyclothymia) are summarized under the label “other specified bipolar and related disorders”.

Given these changes, DSM-5 seems to concur with the idea that there has been an under-recognition of bipolar disorders. However, in return, the obligatory symptoms (gate A criteria) which have to be present to fulfill the criteria for a hypomanic or manic episode have been specified. While in the past only a distinct period of abnormally and persistently elevated, expansive or irritable mood was necessary, these symptoms now have to be present in combination with persistently increased (goal-directed) activity or energy, most of the day, nearly every day. While some disagree with this step, for understandable reasons (Angst et al. 2011,2012), we feel that this is a wise approach, in particular with regard to the diagnosis of bipolar II disorder. Why do we feel this way?

Bipolar II disorder is the only psychiatric disorder which is typically characterized by the absence of the critical constituent, i.e. the hypomanic episode, at the time of diagnosis. The diagnosis is most often assigned to young patients presenting with a (first) major depressive episode. In these cases, diagnosis is exclusively based on psychiatric history taken, not on current psychopathological assessment by the psychiatrist. However, any retrospective recall is prone to recall bias. This may be even more significant during a depressive episode. In addition, with a hypomanic episode, there is a condition at stake which, by definition, is insufficiently severe to cause (significant) impairment in social or occupational functioning. In fact, it may even accompany a heightened level of creativity. Consequently, a hypomanic episode is frequently judged by the patient as being ego-syntonic. Therefore, finding out whether, at some point in the past, there has been a change in mood, associated with an unequivocal change in functioning, that is uncharacteristic of the individual when not symptomatic may significantly depend on the information provided by others, such as close friends, relatives or partners. Unfortunately, the information provided by these others is rarely gathered in scientific studies involving issues related to making the diagnosis of bipolar II disorder. A change in mood in the direction of elevated mood, for example, is primarily a subjective experience, not necessarily associated with an unequivocal change in functioning - and thereby not necessarily easily accessible to others. In contrast, (hypo)mania-associated change in mood, by definition, has to be accompanied by an unequivocal change in functioning. Therefore, a further specification of the change in mood with which (hypo)mania is associated is clearly needed. From a clinical point of view, this change in mood is well captured by the term “hyper” (which is, incidentally, the screening question for (hypo)mania in SCID for DSM-IV). Being hyper invariably includes being highly energetic. Therefore, from a clinician and DSM perspective, it is a completely logical and consistent step to formally add increase in (goal-directed) activity/energy to the change in mood as a gate A criterion in DSM-5.

While diagnoses may have diverse functions (e.g. as a tool for communication about features/symptoms or as justification for claiming of benefits and reimbursements in the healthcare system), informing treatment decisions is one of the most crucial (Cuthbert and Insel 2013). In the clinical example described above (a young patient with a first major depressive episode), whether a diagnosis of major depressive disorder or bipolar II disorder is made will have a large and significant impact on the future treatment, and especially the long-term treatment. According to current treatment guidelines, a young patient with a first major depressive episode in the context of a major depressive disorder will likely be treated with an antidepressant for a period of 6 to 12 months, depending on a variety of (clinical) variables, such as severity of the depressive episode or family history (Bauer et al. 2013). In contrast, a patient with the diagnosis of bipolar depression will probably be treated with either quetiapine or a combination of an antidepressant and a prophylactic antimanic agent (sometimes referred to as a “mood stabilizer”) (Pfennig et al. 2012). Quetiapine or the mood stabilizer, if effective, will be given until further notice. One of the criteria for efficacy will be the prevention of new hypomanic/manic episodes. Now, if we think of diagnostic criteria as a “type of test for the underlying, etiologically defined, illness”, lowering the diagnostic threshold for bipolar disorders, as proposed by some, will increase the probability of false positives and reduce the probability of false negatives, and vice versa (Zimmerman 2012). With regard to our example, a young patient with a major depressive episode who is falsely diagnosed with a bipolar disorder (whereas in reality he/she is suffering from unipolar depression) will be treated with a prophylactic antimanic agent (mood stabilizer) and this treatment may continue indefinitely as one of the criteria for efficacy will be the prevention of new manic episodes - which the patient will not develop as he/she is, in reality, suffering from unipolar depression. In contrast, if a patient with a major depressive episode is falsely diagnosed with major depressive disorder, whereas, in reality, the patient is suffering from bipolar II disorder (as the most probable case), the patient will be treated with an antidepressant (which, according to a recent expert survey, is a legitimate treatment option for bipolar II disorder) (Pacchiarotti et al. 2013). If the patient does not respond to the antidepressant, it will be augmented with lithium, quetiapine, aripiprazole or olanzapine (Bauer et al. 2013). Lithium, quetiapine, aripiprazole and olanzapine are all prophylactic antimanic agents (mood stabilizers), and the combination of an antidepressant and a prophylactic antimanic agent is a viable treatment option for long-term treatment in patients with bipolar II disorder (Pacchiarotti et al. 2013). Alternatively, if such a patient develops a hypomanic or manic episode during antidepressant monotherapy, which continues for a substantial period of time after cessation of the antidepressant, a diagnosis of bipolar disorder will be made according to the current DSM-5 criteria, and the individual will be treated accordingly. Therefore, in terms of the requirement to “do no harm”, the consequences of being falsely diagnosed with bipolar disorders tend to be more severe than those of being falsely diagnosed with major depressive disorder (Frances and Jones 2012). In addition, if the goal of diagnosis is not only to inform current treatment decisions but also to contribute in developing future treatment options, having patients with falsely diagnosed bipolar disorders in genome-wide association studies (GWAS) may cloud statistically significant associations - and thereby prohibit the development of tailored personalized treatment options, based on the findings of these GWAS, for patients with bipolar disorders (Schulze 2010).

In summary, in our view, the DSM-5 criteria nicely specify what is currently understood by the diagnosis of “bipolar disorders” (with the current treatment options based upon these definitions) and at the same time allow further exploration of the nature of disorders (e.g. in terms of treatment response) which, at this point in time, have to be referred to as disorders related to bipolar disorders.
